# Effects of heterologous human tau protein expression in yeast models of proteotoxic stress response

**DOI:** 10.1111/cns.14304

**Published:** 2023-06-21

**Authors:** Klara Zubčić, Dina Franić, Mihaela Pravica, Patrick R. Hof, Goran Šimić, Mirta Boban

**Affiliations:** ^1^ Croatian Institute for Brain Research, University of Zagreb School of Medicine Zagreb Croatia; ^2^ Nash Family Department of Neuroscience, Ronald M. Loeb Center for Alzheimer's Disease Friedman Brain Institute, Icahn School of Medicine at Mount Sinai New York New York USA

**Keywords:** chronological aging, proteasome, protein aggregation, protein homeostasis, protein interaction assay, protein quality control, toxicity, yeast

## Abstract

**Background:**

The primary histological characteristic of Alzheimer's disease is the presence of neurofibrillary tangles, which are large aggregates of tau protein. Aging is the primary risk factor for the development of Alzheimer's disease, however, the underlying causes of tau protein aggregation and toxicity are unclear.

**Aims:**

Here we investigated tau aggregation and toxicity under the conditions of compromised protein homeostasis.

**Methods:**

We used heterologous expression of human tau protein in the unicellular eukaryote yeast *Saccharomyces cerevisiae* with evolutionarily conserved protein quality control pathways and examined tau‐dependent toxicity and aggregation using growth assays, fluorescence microscopy, and a split luciferase‐based reporter NanoBiT.

**Results:**

Tau protein expressed in yeast under mild proteotoxic stress, or in mutants with impaired pathways for proteotoxic stress response, did not lead to synthetic toxicity or the formation of obvious aggregates. Chronologically old cells also did not develop observable tau aggregates. Our examination of tau oligomerization in living cells using NanoBiT reporter suggests that tau does not form significant levels of oligomers under basal conditions or under mild proteotoxic stress.

**Conclusion:**

Together our data suggest that human tau protein does not represent a major burden to the protein quality control system in yeast cells.

## Abstract 2

When expressed in yeast *Saccharomyces cerevisiae* cell model under the conditions of compromised protein homeostasis, human tau protein does not form visible aggregates or result in toxicity.

## 1 INTRODUCTION

One of the most important neuropathological changes in Alzheimer's disease (AD) is the accumulation of tau protein aggregates in the form of neurofibrillary tangles in neurons and glial cells.^1^ Affected brain areas progressively lose neurons, however, the exact causes of tau protein aggregation and toxicity are unclear.^2^, ^3^ Under physiological conditions, tau is a highly soluble protein primarily located in the neurons in the central nervous system, where it binds and stabilizes axonal microtubules.^1^, ^4^ The exact physiological function of tau is still not completely clear, however, it has been found that tau regulates axonal microtubule polymerization via a highly dynamic interaction with microtubules, termed the “*kiss and hop*” interaction.^5^ In the adult human brain tau is expressed in six splicing isoforms that differ in the N‐terminal insertions encoded by exons‐2 and ‐3, and in the C‐terminal microtubule‐binding domain encoded by exon‐10.^1^ Tau is a phosphoprotein that can be phosphorylated at multiple sites by various protein kinases, including GSK3β, MAPK13, and AMP‐activated protein kinase.^1^ Phosphorylation negatively regulates the ability of tau to stimulate microtubule assembly. Tau from the brains of AD patients is hyperphosphorylated, which suggested that phosphorylation plays a role in tau pathology and aggregation, however, it appears that tau aggregation does not require abnormal phosphorylation once the process has been initiated.^1^ In AD, tau accumulates in neuronal soma, where it forms paired helical filaments and further aggregates into neurofibrillary tangles (NFTs).^1^, ^6^ In addition to losing its physiological function, pathologically altered tau exhibits a toxic gain‐of‐function, however, the underlying pathways of neurotoxicity are not entirely clear. Although NFT is the most prominent neuropathological feature of AD, neurotoxicity is at least partially caused by tau oligomers and early‐stage aggregates.^7^, ^8^, ^9^ Accordingly, tau oligomers were found elevated in post‐mortem samples of the AD brain.^10^

Under normal circumstances, cells maintain protein homeostasis and prevent the accumulation of aberrant proteins by a protein quality control system, a network of evolutionarily conserved pathways, including selective protein degradation by the proteasome.^11^, ^12^ Accumulation of misfolded proteins in the cell is a condition that represents proteotoxic stress.^13^ Impaired selective protein degradation is one of the possible underlying causes of several neurodegenerative diseases characterized by protein aggregation.^14^ Since the ability of the cell to maintain protein homeostasis decreases with aging,^15^ impaired protein homeostasis is considered a possible factor in the development of tauopathies.^16^, ^17^ Furthermore, excessive amounts of misfolded proteins in the endoplasmic reticulum (ER) lead to ER stress and activates pathways commonly referred to as the unfolded protein response (UPR).^18^, ^19^, ^20^ Recent studies have indicated a connection between ER stress and AD.^21^, ^22^, ^23^, ^24^

To better understand the factors contributing to tau protein aggregation and toxicity, here we studied human tau protein expressed in yeast *Saccharomyces cerevisiae*, a single‐cell eukaryote. Taking advantage of yeast as an organism suitable for genetic manipulations, earlier studies examined the impact of heterologous expression of human proteins associated with the development of neurodegenerative diseases (such as amyloid‐β,^25^, ^26^ α‐synuclein,^27^, ^28^ huntigtin^29^ and c9orf72^30^, ^31^) on yeast cell growth, and factors contributing to the toxicity of these proteins have been identified. Several earlier reports have studied human tau in yeast^25^, ^32^, ^33^, ^34^, ^35^, ^36^ and the suitability of yeast to study tau has been reviewed.^37^, ^38^ In yeast, tau can be phosphorylated by the ortholog of human GSK3β kinase Mds1, whose activity is negatively regulated by Pho85, an ortholog of human Cdk5 kinase.^25^, ^32^ Tau aggregates present in the brains of patients with AD are characterized by the insolubility in the detergent sarkosyl.^39^ In previous studies, yeast cells expressing human tau protein were biochemically analyzed for the protein solubility in sarkosyl, and a sarkosyl‐insoluble tau fraction corresponding to around 0.02% of total tau protein was detected in the wild‐type strain, while it increased to around 0.4% in the *pho85D* mutants that exhibit increased phospho‐tau levels.^25^, ^32^ In this study, our approach was to use two methods that allow monitoring of tau protein aggregation in living cells, via fluorescent microscopy of tau fused to fluorescent proteins, and by a split luciferase assay.

Since there is no yeast ortholog of tau, yeast is a suitable model for investigating toxic gain‐of‐function. Earlier studies indicated that tau protein expressed in yeast is not toxic under basal conditions.^34^, ^40^ Tau protein toxicity in yeast has been demonstrated in a strain with a mutation in *ESS1*, a gene that codes for peptidyl‐prolyl *cis*–*trans* isomerase that is homologous to the enzyme Pin1 in mammalian cells.^33^ Additionally, two studies investigated a potential synergistic effect of alpha‐synuclein on tau toxicity and aggregation in yeast,^34^, ^35^ however, it is not entirely clear whether this is the effect of different alpha‐synuclein expression levels or the presence of tau.^34^, ^35^ It has not been known whether tau becomes toxic under the conditions of compromised protein homeostasis, such as when cells encounter an increased load of misfolded proteins, or in cells that are deficient in proteotoxic stress response.

Many protein quality control pathways, including selective protein degradation by the ubiquitin‐proteasome system (UPS) and some UPR pathways, are evolutionarily conserved from yeast to humans.^41^, ^42^ Key components of the yeast UPR are Ire1, a sensor for unfolded proteins, and Hac1, a transcriptional factor that activates genes that suppress the consequences of proteotoxic stress.^43^ In yeast, transcription factor Rpn4 plays a critical role in regulating proteasome levels by controlling the expression of genes involved in proteasome biogenesis and assembly. Under normal conditions, Rpn4 is rapidly degraded by the proteasome, however, in response to stress that overloads the proteasome with degradation substrates, Rpn4 is stabilized and activates transcription of proteasomal genes, thus increasing the abundance of the proteasomes.^44^ Mutants lacking Rpn4 exhibit lower levels of proteasomes under basal conditions and an increased susceptibility to proteotoxic stress.^45^ Together Rpn4‐ and Ire1/Hac1‐dependent pathways represent two main proteotoxic stress response pathways in yeast.^46^

Here, using heterologous expression in yeast *Saccharomyces cerevisiae*, we investigated human tau protein toxicity and aggregation under the conditions of compromised protein homeostasis and in mutants deficient in proteotoxic stress response. Our results indicate that mild proteotoxic stress does not promote toxicity and aggregation of tau in yeast cells.

## 2 METHODS

### 2.1 Yeast growth media

Standard yeast media, such as liquid and solid yeast extract‐peptone‐dextrose medium and ammonia‐based synthetic complex dextrose (SCD) medium containing 2% d‐glucose were prepared as described.^13^ Where indicated, 2% galactose was used in the SC medium. Cells were grown at 30°C unless indicated otherwise.

### 2.2 Yeast strains

Yeast *S. cerevisiae* strains were based on BY4741 (Euroscarf) and are listed in Table 1. For details on yeast strain construction, see Appendix S1.

**TABLE 1:** Yeast strains used in the study.

Yeast strain	Genotype	Reference
BY4741	*MATa; his3Δ1; leu2Δ0; met15Δ0; ura3Δ0*	Euroscarf (Germany)
BY4742	*MATα; his3Δ1; leu2Δ0; lys2Δ0; ura3Δ0*	Euroscarf (Germany)
*rpn4∆*, Y03716	*BY4741; MATa; his3Δ1; leu2Δ0; met15Δ0; ura3Δ0; YDL020c::kanMX4*	Euroscarf (Germany)
*ire1∆*, Y01907	*ura3Δ0; leu2Δ0; his3Δ1; met15Δ0; ire1Δ::kanMX4*	Euroscarf (Germany)
*hac1∆*, Y05650	*ura3Δ0; leu2Δ0; his3Δ1; met15Δ0; hac1Δ::kanMX4*	Euroscarf (Germany)
BY4741_Nup49_mScarlet	*BY4741; MATa; his3Δ1; leu2Δ0; met15Δ0; ura3Δ0;YGL172W::Nup49‐mScarlet‐URA3*	This study

### 2.3 Plasmids

The cDNA sequence of the 2N4R isoform of the wild‐type human tau protein was optimized for the expression in yeast by replacing the least frequently used codons with more frequent ones (the sequence is available in the Appendix S1) and cloned into centromeric plasmids. All plasmids are listed in Table 2. For details on plasmid construction, see Appendix S1.

**TABLE 2:** Plasmids used in the study.

Plasmid	Description	Reference
pKZ04	PTDH3‐tau‐2xHA‐linker‐SmBiT‐Tsynht8, *URA3*, CEN	This study
pKZ05	PTDH3‐tau‐2xHA‐linker‐SmBiT‐Tsynht8, *LEU2*, CEN	This study
pKZ06	PTDH3‐tau‐V5‐linker‐LgBiT‐Tsynth27, *URA3*, CEN	This study
pKZ07	PTDH3‐tau‐V5‐linker‐LgBiT‐Tsynth27, *LEU2*, CEN	This study
pKZ08	PTDH3‐tau‐2xHA‐SmBIT	This study
	PTDH3‐tau‐V5‐LgBiT	
	*URA3*, CEN	
pKZ10	PCUP1‐tau‐TCYC1, *LEU2*, CEN	This study
pKZ11	PCUP1‐tau‐TCYC1, *URA3*, CEN	This study
pKZ12	PGAL1‐tau‐TCYC1, *URA3*, CEN	This study
pKZ15	PTDH3‐TDH3‐V5‐LgBiT, *URA3*, CEN	This study
pKZ24	PTEF1‐tau‐sfGFP, *URA3*, CEN	This study
pKZ35	PTDH3‐tau‐HA‐SmBIT	This study
	PTDH3‐tau‐V5‐LgBiT	
	*URA3*, CEN	
pKZ37	PPIR3‐tau‐sfGFP, *URA3*, CEN	This study
pKZ41	PPIR3‐Tau, *URA3*, CEN	This study
pKZ51	PPIR3‐NeonGreen‐tau, *URA3*, CEN	This study
pMB292	PTEF1, *URA3*, integrative	Mirta Boban laboratory
pMB152	PTEF1, *URA3*, *CEN*	Mirta Boban laboratory
pMB211	PPIR3, *URA3*, *CEN*	Mirta Boban laboratory
pXP722	PGAL1, *URA3*, CEN	Gift from Nancy DaSilva^73^ (Addgene plasmid #46056; http://n2t.net/addgene:46056; RRID:Addgene_46056)
pXP731	PCUP1, *LEU2*, CEN	Gift from Nancy DaSilva^73^ (Addgene plasmid #46057; http://n2t.net/addgene:46057; RRID:Addgene_46057)
pXP732	PCUP1, *URA3*, CEN	Gift from Nancy DaSilva^73^ (Addgene plasmid #46058; http://n2t.net/addgene:46058; RRID:Addgene_46058)
pRS316	*URA3*, CEN	^74^
pRS315	*LEU2*, CEN	^74^
pCA1016	SmBiT‐HA‐sfGFP‐NLS	Gift from Claes Andreasson
pFA6a‐link‐ymScarletI‐URA3	ymScarletI, yeast tagging vector	Gift from Bas Teusink^75^ (Addgene plasmid #168055; http://n2t.net/addgene:168055; RRID:Addgene_168055)

Abbreviations: CEN, centromeric yeast plasmid; PTEF1/PTDH3/PPIR3/PCUP1/PGAL1, promoters of respective genes; TCYC1, *CYC1*‐gene terminator; *URA3*/*LEU2*, selection markers.

### 2.4 Growth‐based assay of cell viability

Cells were grown to the logarithmic growth phase and decimal dilutions of the culture were inoculated onto a solid medium, incubated at 30°C unless indicated otherwise, and photographed after 3 days.

### 2.5 Microscopy

Cells were grown in SCD, fixed in 0.8% formaldehyde for 10 min, centrifuged, and washed twice in phosphate‐buffered saline (PBS), the pellet was resuspended in PBS, and suspension was dropped onto coverslips coated with concanavalin‐A (Sigma‐Aldrich). Images were acquired using Olympus‐BX53 microscope equipped with Olympus‐DP74 digital camera and an Olympus 100×, oil‐immersion 1.4 NA objective.

### 2.6 Luminescence measurements

Luminescence was measured in living cells grown in SCD using Glomax Explorer microplate reader (Promega) and NanoGlo luciferase assay (Promega), according to the manufacturer's protocol. Luciferase substrate NanoGlo was diluted 1:100 in dilution buffer and this solution was added to the cells in a ratio of 1:10. Bioluminescence arbitrary units were defined as the relative light units of 1 × 10^7^ cells.

### 2.7 Immunoblotting analysis

Cells were grown in SC at 30°C unless indicated otherwise, to an optical density at 600 nm (OD_600_) of 0.8–1.0 and pelleted by centrifugation. Total protein was extracted as described previously.^47^ Briefly, cells were harvested by centrifugation, resuspended in 100 μL distilled water, added 100 μL 0.2 M NaOH, incubated for 5 min at room temperature, pelleted at 13,000 rpm for 5 min, and resuspended in 50 μL sample buffer (0.06 M Tris–HCl pH 6.8, 5% glycerol, 2% SDS, 4% β‐mercaptoethanol, 0.0025% bromophenol‐blue). Proteins were denatured at 95°C for 5 min and resolved by SDS‐PAGE. Immunoblotting was performed by antibodies: anti‐tau (MA5‐12808, RRID: AB_10980631, Thermo Fisher Scientific, 1/1000), anti‐V5 (RRID: AB_2556564, Thermo Fisher Scientific, 1/5000), anti‐HA (12CA5, Egon Ogris laboratory, Max Perutz Labs, Vienna, Austria, 1/1000), anti‐Pgk1 (22C5, RRID: AB_2546088, Invitrogen, 1/20,000), HRP‐anti‐mouse (RRID: AB_330924, Cell Signaling Technology, 1/1000). Signal was detected and quantified by imager (ChemiDoc XRS+, BioRad Laboratories), and normalized to Pgk1 protein or to the total protein, using a stain‐free method (BioRad Laboratories).

### 2.8 Statistical analysis

Statistical analysis was done using GraphPad Prism 8. To ascertain the normality of the data distribution within individual groups, the D'Agostino‐Pearson test was employed. For data sets following a normal distribution, parametric tests including the two‐tailed *t* test, anova, and the post hoc Tukey test were utilized. Conversely, for data not adhering to a normal distribution, we implemented the non‐parametric Mann–Whitney test. *α* = 0.05 was set as the level of statistical significance for all tests.

## 3 RESULTS

### 3.1 Human tau protein expression in yeast cells under the conditions of proteotoxic stress and in mutants compromised for proteotoxic stress response does not lead to toxicity

To investigate tau‐induced protein toxicity and aggregation in yeast *Saccharomyces cerevisiae*, in the first step, a gene encoding wild‐type human tau, isoform 2N4R consisting of 441 amino acids, was placed under the control of inducible *CUP1* and *GAL1* gene promoters. The longest tau isoform 2N4R was also used in the previous studies of tau in yeast.^25^, ^32^, ^33^, ^34^, ^35^, ^36^ Taking into consideration that the pathology of the most common, sporadic late‐onset AD is not associated with MAPT mutations, we conducted our research using wild‐type tau.

**FIGURE 1:**
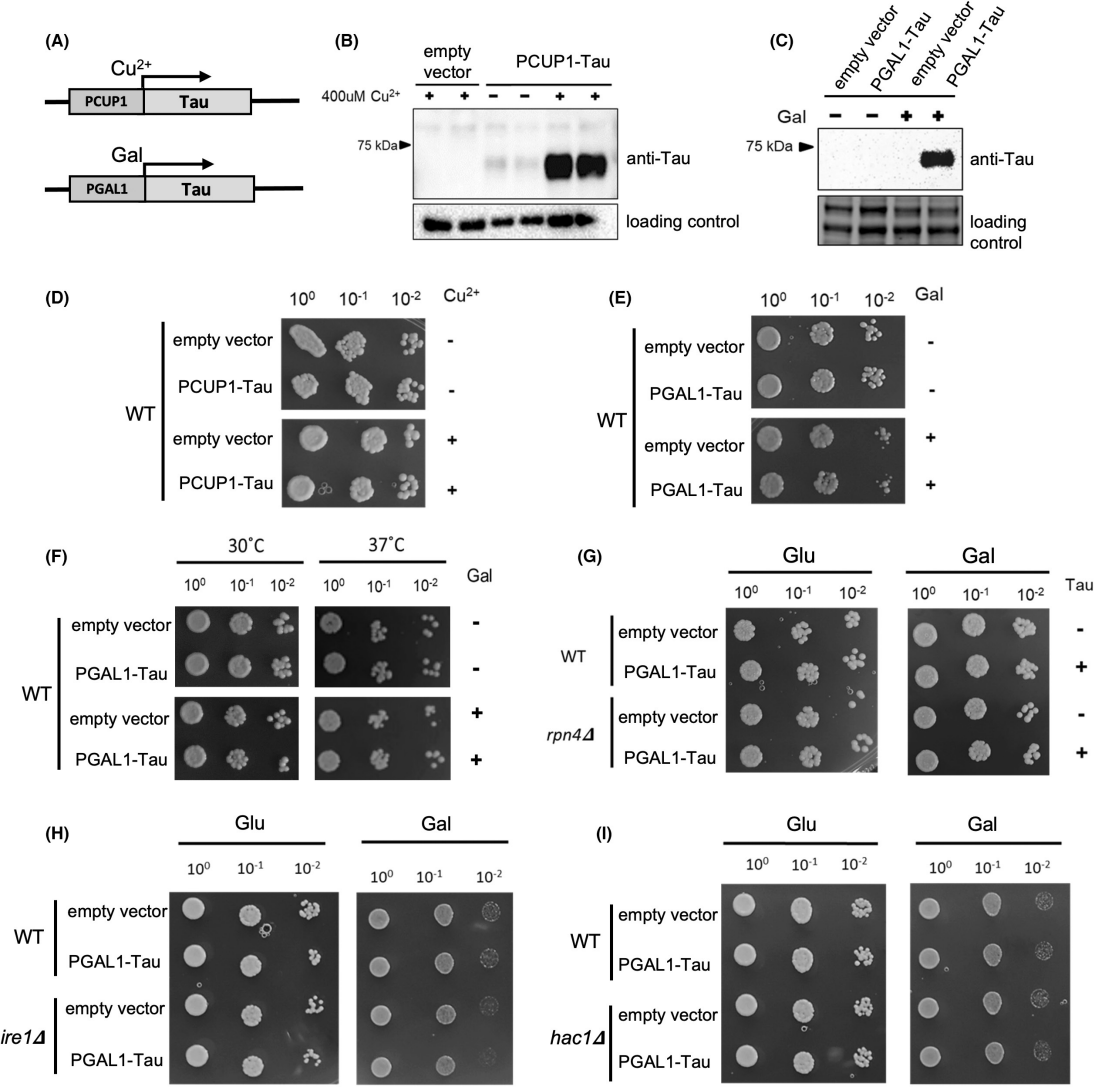
Effect of heterologous human tau protein expression on yeast cell viability in control and *rpn4Δ* and UPR mutants. (A) Diagrams of PCUP1‐tau and PGAL1‐tau constructs for inducible tau protein expression from *CUP1* or *GAL1* gene promoters (PCUP1, PGAL) via copper ion and galactose induction, respectively. (B, C) Inducible human tau protein expression in yeast cells was examined using immunoblots. Yeast strain BY4741 transformed with an empty vector (pXP731) or PCUP1‐tau (pKZ10) (B) or PGAL1‐tau (pKZ12) (C) were grown to the logarithmic growth phase and incubated for 2 h in the presence of 400 μM CuSO_4_ (B) or galactose‐based medium (C), as indicated. The total cell lysate was immunoblotted with an anti‐tau antibody (Tau5). As a loading control, Bio‐Rad stain‐free technology (B) or Pgk1 (C) were used. Tau protein is only expressed at a significant level when cells are stimulated with CuSO_4_ and galactose. In the absence of CuSO_4_, cells with PCUP1‐tau show low tau expression. (D–I) Cell viability tests based on cell growth. The cells were grown to the logarithmic growth phase, and the culture was inoculated onto a solid medium in decimal dilutions. (D, E) Strains from (B) and (C) were inoculated onto solid media with and without 400 μM CuSO_4_ (D) or galactose or glucose‐based media (C), respectively. After 3 days of incubation, no growth delays of cells grown under inducible conditions for tau expression were detected by dot spot. (F) Strain as in (C) was inoculated onto a galactose or glucose‐based solid medium and incubated at 30°C (optimal growth temperature) or 37°C (mild heat shock). After 3 days of incubation at 37°C, no growth delay of cells grown under inducible conditions for tau expression (+Gal) was detected by dot spot. (G) On galactose or glucose‐based solid media, the yeast deletion mutant strain *rpn4Δ* was inoculated after transformation with an empty vector (pXP722) or PGAL1‐tau (pKZ12). After 3 days of incubation, no growth delays of *rpn4Δ* cells grown under inducible conditions for tau expression (+Gal) were observed by the dot spot. (H, I) Yeast deletion mutant strains *ire1∆* and *hac1∆* transformed with an empty vector (pXP722) or PGAL1‐tau (pKZ12) were grown in the same manner as in (F). After 3 days of incubation, no growth delays were observed in *ire1∆* (H) and *hac1∆* (I) mutants grown under inducible conditions for tau expression (+Gal) as measured by the dot spot.

Expression of tau expression from the *CUP1* and *GAL1* promoters was induced by copper (II) sulfate and galactose, respectively (Figure 1A). Immunoblot analysis showed protein bands of the appropriate size that were present only under inducing conditions, demonstrating the inducible expression of the tau protein in yeast (Figure 1B,C). Next, we tested whether the expression of human tau protein in yeast cells leads to toxicity, by using a standard growth‐based assay. The analysis showed that tau expression does not affect the viability of wild‐type yeast cells (Figure 1D,E), which is in accordance with a previously published study that used constitutive tau expression from *TPI1* promoter.^36^

The expression of human tau protein in wild‐type yeast cells grown under basal conditions did not result in toxicity (Figure 1D,E), however, it is possible that the protein quality control system in the wild‐type cells compensates for the effects of tau protein accumulation, and that tau may be toxic under the conditions of compromised protein homeostasis. To test whether tau protein expression leads to a synthetic effect on cell viability when combined with the proteotoxic stress, we exposed cells to a mild heat stress of 37°C, which leads to a higher burden of misfolded proteins and protein aggregates.^48^ Induced tau protein expression did not affect cell growth at 37°C, neither compared to cells grown at 30°C nor to cells that did not express tau (Figure 1F), which is in concert with the data from a previous study.^34^ Next, we expressed tau in the *rpn4Δ* deletion mutant that exhibits lower levels of proteasomes and an increased susceptibility to proteotoxic stress.^46^ By comparing the growth of *rpn4Δ* mutant and wild‐type cells expressing tau from galactose‐inducible promoter we did not detect difference in cell viability (Figure 1G). Taken together, these results suggest that tau protein does not lead to synthetic toxicity in combination with mild proteotoxic stress or downregulated proteasome levels.

When subject to the accumulation of misfolded proteins in the ER (ER‐stress), *ire1Δ* and *hac1Δ* deletion mutants with non‐functional UPR exhibit slower growth.^47^, ^49^, ^50^, ^51^ To test if the expression of tau protein in yeast cells results in ER‐stress and UPR activation, *ire1Δ* and *hac1Δ* deletion mutants expressing tau from an inducible *GAL*‐promoter were examined for growth. Compared to the wild type, UPR‐mutants *ire1Δ* and *hac1Δ* expressing tau exhibited no growth delay (Figure 1H,I), suggesting that expression of tau protein in yeast cells does not induce the UPR. The result is in concert with a previous study in which no evidence for the activation of UPR was found in a transgenic mouse model expressing mutant tau,^52^ although UPR markers have been found increased in AD brains, in neurons with the diffuse accumulation of tau, suggesting that the UPR is involved in the early stages of tau pathology (reviewed in Ref. [24]).

### 3.2 Yeast cells under proteotoxic stress and during chronological aging do not form obvious aggregates of human tau protein

**FIGURE 2:**
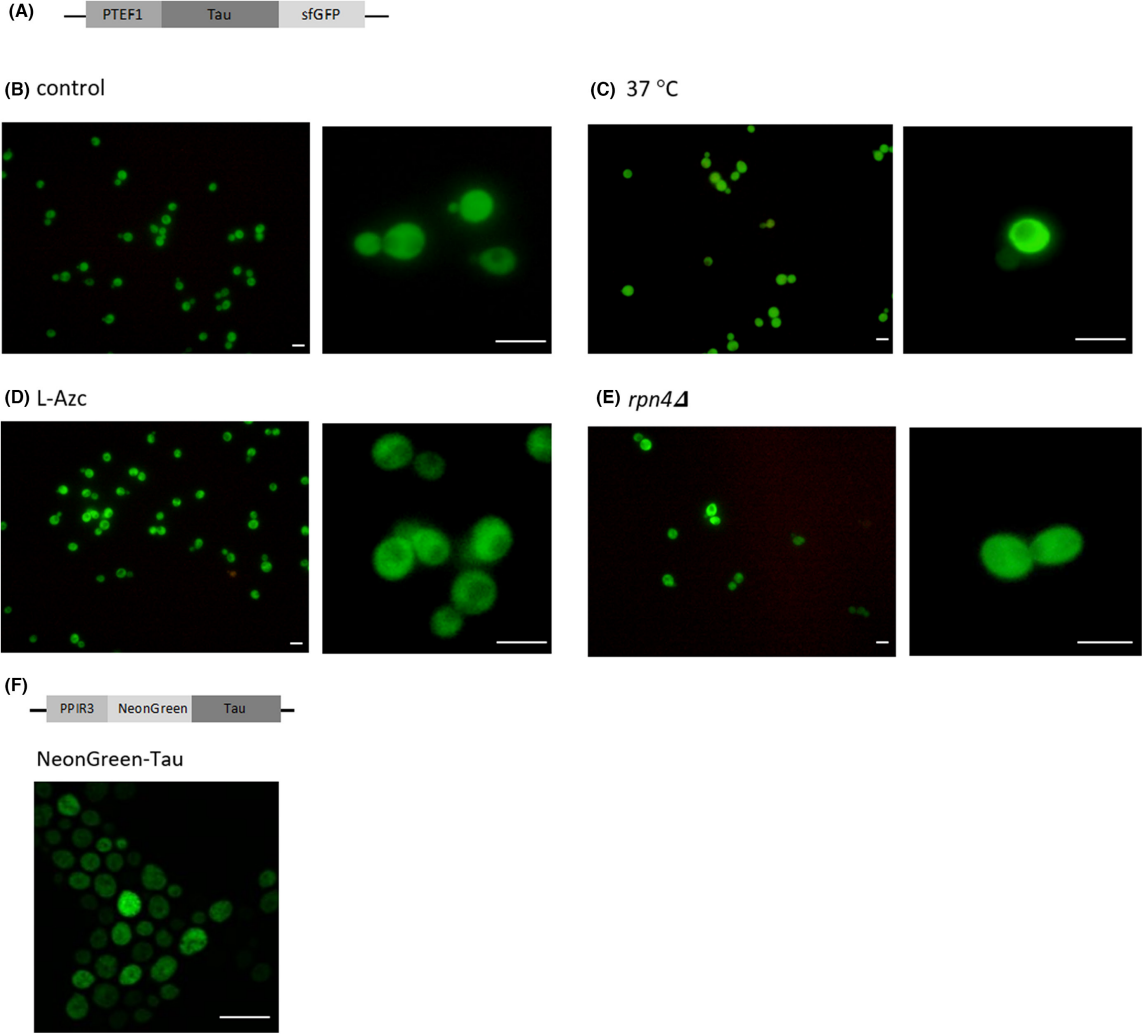
Human tau protein localization in yeast cells under proteotoxic stress and in chronologically aged yeast cells. (A–E) Localization of tau‐sfGFP in cells with exponential growth. (A) Schematic presentation of the PTEF1‐tau‐sfGFP construct (pKZ24). (B) Wild‐type cells (BY4741) transformed with pKZ24 displayed diffuse sfGFP signal when grown under standard conditions and after 2 h of incubation under mild heat stress (37°C) or (C) in the presence of 1 mM L‐azetidine‐carboxylic acid (D). (E) A pKZ24‐transformed *rpn4Δ* mutant strain exhibited a diffuse sfGFP signal. (F) Localization of NeonGreen‐tau in chronologically aged wild‐type cells. Schematic presentation of the PPIR3‐NeonGreen‐tau construct (pKZ51) is shown. NeonGreen‐tau is diffusely localized in cells from 3‐days old cultures of the wild‐type strain (BY4741 transformed with pKZ51). Scale bar = 10 μm.

To investigate the localization of human tau protein expressed in yeast cells by fluorescent microscopy, we genetically fused superfolder GFP (sfGFP) to the C‐terminal end of tau and placed it under the control of a constitutive *TEF1*‐promoter. Tau‐sfGFP was diffusely localized in the cytoplasm of logarithmically growing wild‐type yeast cells (Figure 2A), which is in accordance with previous findings^33^ and indicates that tau in yeast cells does not form aggregates visible by fluorescent microscopy. Next, we investigated whether tau forms visible aggregates in cells subject to proteotoxic stress. To this end, the localization of tau‐sfGFP was analyzed in cells incubated at an elevated temperature of 37°C (Figure 2B), in cells incubated with l‐azetidine‐carboxylic acid (L‐AzC), a toxic analog of l‐proline that leads to protein misfolding (Figure 2C), and in the *rpn4Δ* mutant with downregulated proteasomes (Figure 2D). Under those conditions, tau‐sfGFP localization in the cytoplasm was diffuse, similar to that in wild‐type yeast cells, indicating that proteotoxic stress does not result in the formation of visible tau aggregates.

Age‐dependent protein aggregation is an evolutionarily conserved phenomenon associated with many neurodegenerative diseases^53^, ^54^; therefore, we examined the influence of cellular aging on tau protein aggregation by expressing fluorescently labeled tau in a yeast model of chronological aging. Tau was expressed from the *PIR3*‐gene promoter that becomes active after diauxic shift.^55^ Cells were grown in liquid culture for several days without media change, resulting in the entry of cells into a quiescence‐like state and chronological aging.^56^ Yeast cell culture enters the stationary phase on the second day of growth on a synthetic medium.^57^ In our experiments, tau protein localization was examined in 3 days old cell culture, which is an early phase of chronological aging. In approximately 10% of the cells from the 3 days old culture, tau‐sfGFP formed punctate inclusions that were localized perinuclearly (Figure S1A). To examine whether the observed tau‐sfGFP aggregates form independently of the fluorescent reporter fusion, we constructed a fusion protein in which tau is tagged with a different fluorescent protein, NeonGreen, at the N‐terminus. Cells expressing NeonGreen‐tau fusion protein in 3 days old culture showed a diffuse localization and no inclusions were observed (Figure S1B). Therefore, the aggregates of tau‐sfGFP that were observed in 10% of chronologically aged cells (Figure S1A) were likely a consequence of the sfGFP, rather than the tau aggregation. Together the results indicated that tau does not form visible aggregates in chronologically aged yeast cells, at least during the initial stages of chronological aging.

### 3.3 Luciferase‐based tau‐NanoBiT reporter for studying tau protein oligomerization in yeast

**FIGURE 3:**
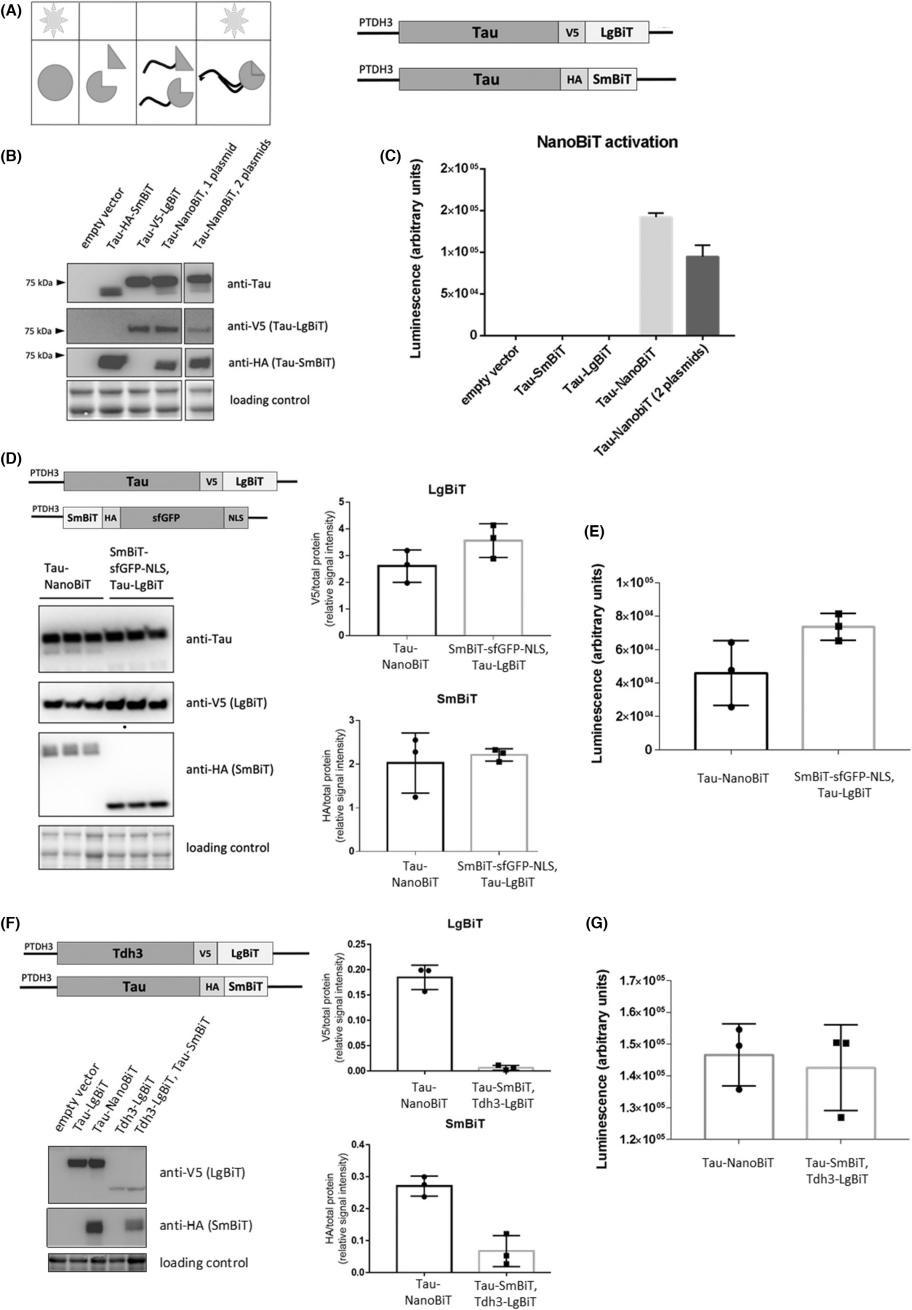
Tau‐NanoBiT reporter for tau–tau interaction detection. (A) Schematic presentation of the NanoBiT (left) and tau‐NanoBiT (right) reporter constructs. Details can be found in the main text. Indicated are the small subunit (SmBiT), large subunit (LgBiT), *TDH3* gene promoter (PTDH3), HA, and V5 epitope tags. (B) Immunoblot analysis of yeast tau‐NanoBiT protein expression. The yeast wild‐type strain (BY4741) was transformed with plasmid pRS316 (empty vector), plasmid pKZ04 (Tau‐SmBiT), plasmid pKZ06 (Tau‐LgBiT), both plasmids pKZ05 and pKZ06 or plasmid pKZ08. (tau‐NanoBiT construct). Total protein lysates were analyzed by western blot using antibodies against tau (tau5) or against epitope tags (anti‐HA, anti‐V5). The last lane was imaged simultaneously from the same blot. (C) Measurement of the luminescence of living cells expressing NanoBiT constructs, as in (B). Only cells expressing tau fusions to both SmBiT and LgBiT exhibited detectable levels of luminescence. (D–G) Examining the specificity of tau‐tau interactions with the tau‐NanoBiT reporter. (D) Immunoblot analysis of the expression of the tau‐NanoBiT and SmBiT‐sfGFP‐NLS proteins in yeast cells. Wild‐type BY4741 strains expressing tau‐NanoBiT (pKZ08) or SmBiT‐sfGFP‐NLS (pCA1016) and tau‐LgBiT (pKZ06) were used to prepare total protein lysates. Lysates were analyzed by immunoblot using antibodies against tau (tau5) and epitope tags (HA and V5). There are three independent replicates for each sample. The data were analyzed using a t‐test with two tails. On the graphs, the signal from the immunoblot on the left is quantified. A schematic illustration of the tested construct SmBiT‐sfGFP‐NLS is shown. Details can be found in the main text. (E) Luminescence measurement of living cells expressing NanoBiT as described in (D). *p* = 0.1 was determined using a two‐sided Mann‐Whitney test on the data. (F) Immunoblot analysis of the expression of the tau‐NanoBiT and Tdh3‐LgBiT proteins in yeast cells. Total protein lysates from the wild‐type strain (BY4741) expressing tau‐NanoBiT (pKZ35) or TDH3‐LgBiT and Tau‐SmBiT (pKZ15 and pKZ05) were analyzed by western blot with antibodies against tau (tau5) and epitope tags (HA and V5). There are three independent replicates for each sample. The data were analyzed using a t‐test with two tails. On the graphs, the signal from the immunoblot on the left is quantified. Presented is a schematic illustration of the construct Tdh3‐LgBiT. Details can be found in the main text. (G) Luminescence measurement of living cells expressing NanoBiT as described in (F). The data were analyzed using the two‐sided Mann‐Whitney test; *p* > 0.9999. All error bars represent the mean ± SD.

To study the factors involved in the tau protein oligomerization, we used a luminescent reporter based on the split luciferase, NanoBiT,^58^ and adapted it for use in yeast (Figure 3). In NanoBiT, luciferase NanoLuc is divided into a small (SmBiT) and a large fragment (LgBiT), that have very low affinity for each other, therefore their interaction, and thus the reconstitution of the luciferase, depends on the ability of the proteins fused with the NanoBiT fragments to interact. We genetically fused NanoLuc fragments to the C‐terminal end of tau (Figure 3A). tau‐SmBiT and tau‐LgBiT were tagged with the epitope tags HA and V5, respectively, to facilitate the distinction of the fragments on immunoblot. To reduce the likelihood that protein fusion will affect tau structure, a flexible Gly‐Ser repeat was inserted between the epitope tags and the luciferase fragments (tau‐HA‐Gly‐Ser‐SmBiT and tau‐V5‐Gly‐Ser‐LgBiT). For simplicity, the constructs are referred to as tau‐SmBiT and tau‐LgBiT in the text. Each fusion was placed under the control of the constitutively active *TDH3* gene promoter and the constructs were integrated into a common plasmid vector in antiparallel directions, to avoid sequence loss due to homologous recombination between the tau sequences. To ensure a moderate level of tau protein expression and thus reduce the likelihood of non‐specific interactions between the luciferase fragments, constructs were placed on a centromeric (CEN) vector. Luminescence measurements showed that the luminescence of cells expressing individually only tau‐SmBiT or only tau‐LgBiT is negligible compared to cells expressing tau‐NanoBiT, i.e. both constructs (Figure 3C). Reporter activation was tested in cells expressing both constructs from the same plasmid (pKZ35) and in cells expressing the two constructs from the two separate plasmids (pKZ6 and pKZ7). Due to a higher level of reporter activation, and for simplicity, in further experiments that require the expression of tau‐NanoBiT (i.e. the expression of both constructs, tau‐LgBiT and tau‐SmBiT) in the same cell, expression from the single plasmid pKZ35 was used.

To test whether the observed activation of the tau‐NanoBiT reporter (Figure 3C) is a result of the specific tau‐tau interaction, or whether it represents a basal level of NanoBiT reporter activation due to the co‐expression and co‐localization of luciferase fragments, we compared luminescence of cells expressing both tau‐LgBiT and tau‐SmBiT (tau‐NanoBiT) with luminescence of cells in which one luciferase fragment (SmBiT or LgBiT) was fused with tau, and the other fragment was fused to an unrelated protein that according to the literature is not likely to interact with tau protein. To this end, SmBiT was fused with sfGFP tagged with the nuclear localization signal (SmBiT‐HA‐sfGFP‐NLS) and co‐expressed with tau‐LgBiT (Figure 3D). As a second control for the specificity of tau‐NanoBiT reporter activation, we co‐expressed the tau‐SmBiT construct with the Tdh3‐LgBiT construct that contains glyceraldehyde‐3‐phosphate dehydrogenase Tdh3 (Figure 3F). For each of the constructs above (Figure 3D,F) we examined the protein expression using immunoblot. In cells co‐expressing tau‐LgBiT and SmBiT‐sfGFP‐NLS, luminescence was not significantly different from in cells expressing tau‐NanoBiT (tau‐LgBiT and tau‐SmBiT) (Figure 3E). Likewise, in cells co‐expressing tau‐SmBiT and Tdh3‐LgBiT, luminescence was similar as in cells expressing tau‐NanoBiT (Figure 3G). The construct in which LgBiT was fused only with the V5 epitope (V5‐LgBiT) was not expressed in the cells, or its level was too low to be detected by Western blotting (results not shown), therefore this construct could not be used as a control for the specificity of the activation of the NanoBiT reporter.

In conclusion, bioluminescence in the wild‐type yeast cells expressing tau‐NanoBiT likely represents a basal level of reporter activation due to the co‐expression and co‐localization of luciferase fragments, suggesting that under the conditions tested, tau does not form oligomers, or the levels of tau oligomers are very low.

### 3.4 Mild proteotoxic stress and deficiency in Rpn4‐dependent proteotoxic stress response do not induce activation of Tau‐NanoBiT reporter in yeast cells

**FIGURE 4:**
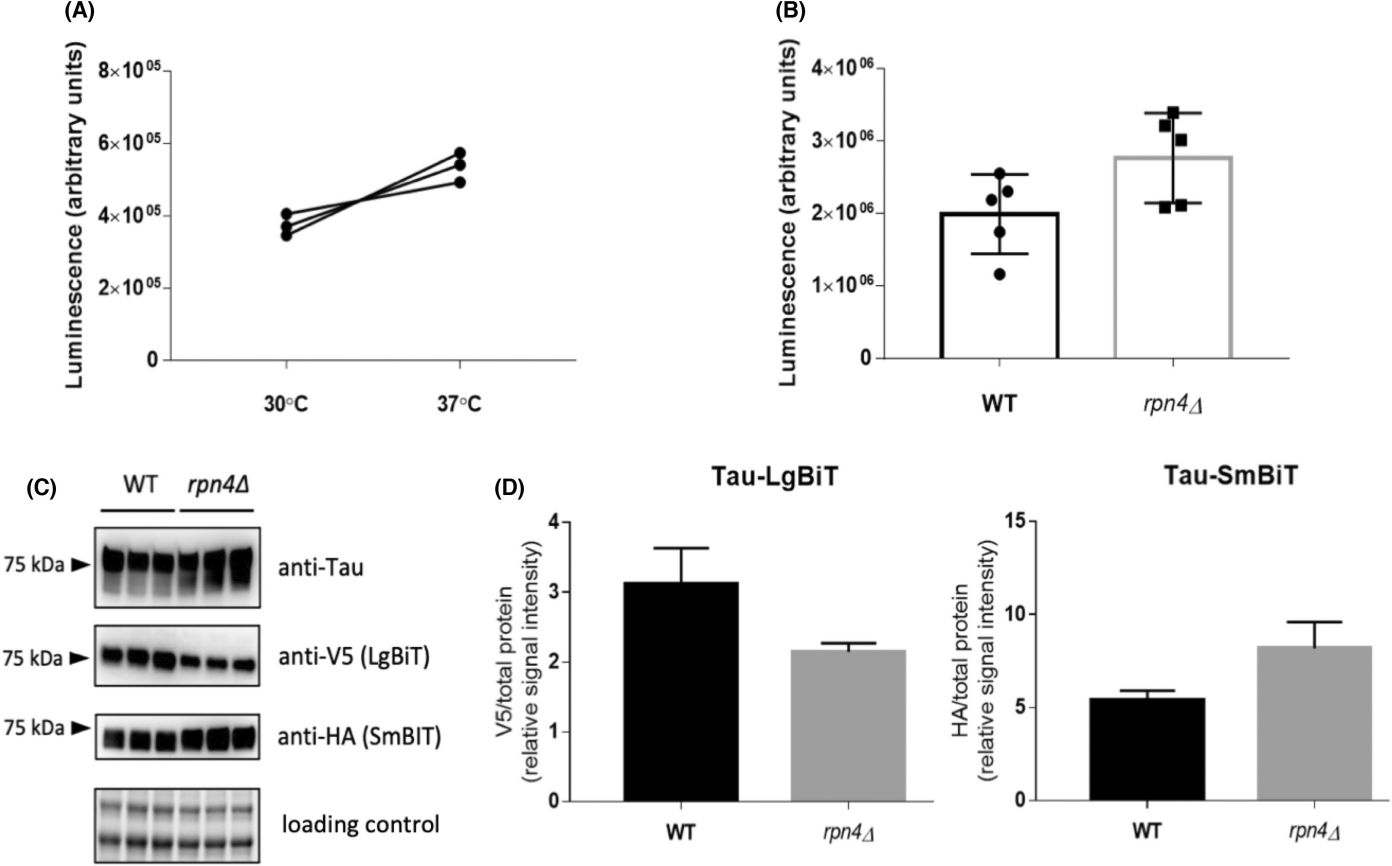
Analysis of the activation of the tau‐NanoBiT reporter in yeast cells under proteotoxic stress and in the *rpn4∆* mutant. (A, B) Measurement of the luminescence of living cells expressing the tau‐NanoBiT reporter (pKZ08). (A) Log‐growing wild‐type cells (BY4741) were grown at 30°C to an OD_600_ of 1.0, and aliquots were incubated for 30 min at 30 or 37°C. After treatment, luminescence was measured at RT immediately. (B) The luminescence of log‐growing wild‐type (BY4741) and *rpn4∆* mutant cells were measured at an OD_600_ of 1.0. (C) Analysis of tau‐NanoBiT (pKZ08) expression by immunoblot in wild‐type (BY4741) and *rpn4∆* cells. Total protein lysates were analyzed by western blot using antibodies against tau (tau5) or against epitope tags (anti‐HA, anti‐V5). There are three independent replicates for each sample. (D) The graphs display the signal quantification from the immunoblot on the left (C). All error bars represent the mean ± SD.

Impaired protein homeostasis and consequent proteotoxic stress are considered possible cause of neurodegenerative diseases.^59^ Therefore we examined the impact of proteotoxic stress on tau protein oligomerization by measuring tau‐NanoBiT reporter activation. When proteotoxic stress was induced by incubating yeast cells at an elevated temperature of 37°C for 30 min, there was no statistically significant increase in reporter activation compared to the cells incubated at 30°C (Figure 4A). Next, we expressed tau‐NanoBiT reporter in cells with *RPN4* gene deletion (Figure 4B). We did not record a statistically significant increase in tau‐NanoBiT in the *rpn4* deletion mutant compared to the wild‐type (Figure 4B). In conclusion, the results suggest that the conditions of mild proteotoxic stress and impaired Rpn4‐dependent regulation of proteasome abundance do not induce tau protein oligomerization under the conditions tested.

## 4 DISCUSSION

One of the hallmarks of aging is a decline in protein homeostasis that manifests in the accumulation of protein aggregates, suggesting that aged cells are not fully capable of controlling the accumulation of aberrant proteins. Compromised protein homeostasis, and consequent proteotoxic stress, has been considered a possible cause of neurodegenerative diseases.^53^, ^59^ For instance, several studies have linked tau protein pathology to the UPS, although it remains unclear whether tau is the cause or a consequence of an impaired UPS.^60^, ^61^, ^62^, ^63^, ^64^

Here we investigated how the increased burden of misfolded proteins and mutations in the proteotoxic stress response pathways influence human tau protein when expressed in yeast. Earlier studies indicated that tau protein is not toxic for yeast cells under basal conditions,^34^, ^35^ however, it has not been known whether tau becomes toxic under the conditions of compromised protein homeostasis, such as when cells encounter an increased load of misfolded proteins. Our approach was to simulate the conditions of proteostasis decline. First, we induced the accumulation of misfolded proteins by using a mild heat shock or growing cells in the presence of a toxic amino acid analog. Second, we disrupted proteotoxic stress response pathways mediated by Rpn4, the regulator of proteasome abundance, and by Ire1‐Hac1‐dependent UPR.^45^ None of the tested conditions had an effect on the growth of tau‐expressing cells. One possibility is that tau does not represent a substrate for the protein quality control system, that is, the cell does not recognize it as an aberrant protein. Alternatively, the tested conditions may represent a moderate stress that the cell successfully compensates for, or bypasses the impaired pathways with alternative mechanisms. In this case, the toxic phenotype may manifest under more severe stress, or in multiple mutants for different protein quality control pathways. Furthermore, a recent study has shown that expression of a cytoplasmic model misfolded protein NBD2* in the *rpn4* mutant did not lead to slower cell growth, while the expression of several membrane misfolded mutant proteins resulted in slower growth of the *rpn4* mutant.^65^ Based on that finding, it is possible that protein quality control mutants other than *rpn4* may have a synthetic phenotype with human tau.

Although NFT is the most prominent neuropathological feature of AD, it appears that the neurotoxicity is at least partially caused by tau oligomers and early‐stage tau aggregates.^44^, ^45^, ^46^ To be able to monitor tau protein oligomerization in living cells without the need for biochemical analyzes of cell lysates, we used a split luciferase reporter. Since a previous study on HEK‐293 cells showed oligomerization of tau that was C‐terminally tagged with the Gaussia luciferase,^66^ we designed tau‐NanoBiT using tau C‐terminal fusions with luciferase fragments. We observed that when both fusion proteins, tau‐LgBiT, and tau‐SmBiT, were co‐expressed in the same cells, their steady‐state protein levels were uneven (Figure 3B). Taking into consideration that the bioluminescence intensity depends on the amount of reconstituted luciferase, the expression level of the fusion proteins in the samples that are compared has been taken into account when interpreting the results (Figures 3 and 4). Uneven levels of two heterologous proteins upon their co‐expression in the same cells is not a phenomenon that is specific for tau‐NanoBiT. For example, co‐expression of tau with other proteins in yeast cells has previously been reported to lead to a lower tau expression.^41^ Furthermore, when using NanoBiT and in the study of Sod1 protein homodimerization in neurons, Oh‐hashi et al observed unequal expression levels of constructs despite the use of the same promoters in Sod1‐LgBIT and Sod1‐SmBiT fusions.^67^ Uneven expression of two heterologous proteins appears to be a common feature when using reporter proteins, not only in luciferase‐based systems but also in fluorescent reporters.^68^

In our experiments, activation of the NanoBiT reporter consisting of one luciferase fragment fused to tau and the other fused to a protein that is not predicted to interact with tau at a considerable level is likely due to non‐specific proximity of the luciferase fragments when co‐expressed in the same cell. However, it has been previously shown that tau interacts with ribosomes in human tissue samples, and shows even stronger connections with ribosomes and ribosome‐binding proteins in AD brain.^69^ Based on those findings, it is also possible that the activation of the control reporters is a result of tau proximity to unrelated proteins during their translation on the ribosome.^70^ Furthermore, although similar levels of tau oligomers have been reported in a split gaussia‐luciferase assay when using N‐ and C‐terminal fusions of tau to luciferase fragments,^66^ it is conceivable that the addition of a NanoBiT fragment to the tau C‐terminal end may interfere with the formation of tau oligomers. Additionally, tau oligomerization in yeast cells may require the use of seeds or aggregation‐prone tau mutants. During the final preparation of this manuscript, a NanoBiT‐tau sensor in mammalian cells that is based on N‐terminal NanoBiT‐tau fusions has been reported, which investigated full‐length wild‐type tau, disease‐associated tauP301L mutant and the aggregation‐prone K18 tau fragment with P301L mutation.^71^ In that study, the luminescence signal of cells expressing NanoBiT fused to wild‐type tau decreased when cells were treated with a microtubule destabilizing agent, suggesting that this basal signal likely reflects the molecular proximity of microtubule‐bound tau, rather than the tau oligomers. In contrast to wild‐type tau, complementation of NanoBiT fused to the tau‐P301L mutant or K18‐P301L was less sensitive to the microtubule destabilizing agent, and the signal could be increased by the addition of seeds of recombinant tau or brain lysates from mouse model of tau pathology.^71^

Taken together, the basal level of tau‐NanoBiT signal detected in yeast cells in our study likely represents molecular proximity of luciferase fragments due to the protein co‐expression. Future studies will test whether the yeast tau‐NanoBiT can be activated above basal levels by using the seeds from recombinant tau or brain lysates and will examine the effects of tau phosphorylation and aggregation‐promoting tau mutations on the reporter activation. Moreover, it would be interesting to see whether proteotoxic stress would have an impact on tau oligomerization and aggregation under those conditions.

## 5 CONCLUSIONS

In conclusion, our results showing that human tau expressed in yeast under mild proteotoxic stress and in mutants with impaired proteotoxic stress response does not affect cell viability suggest that tau does not present a significant burden to yeast protein quality control system, or that other pathways are involved in mitigation of tau expression. Protein homeostasis is maintained by an intricate network of pathways that in many cases overlap or complement each other, therefore tau aggregation and toxicity may arise upon downregulation of more than one pathway, in combination with a more severe stress, or upon addition of a tau seeding agent.^72^ Future research using inducible tau expression and tau‐tau interaction reporters in unbiased genetic screens may discover new factors affecting tau aggregation and toxicity.

## AUTHOR CONTRIBUTIONS

Klara Zubčić: design of the work; acquisition, analysis, and interpretation of data; drafting the work, revising the work critically for important intellectual content. Dina Franić: acquisition of data; revising the work critically for important intellectual content. Mihaela Pravica: acquisition of data; revising the work critically for important intellectual content. Patrick R. Hof: interpretation of data for the work; revising the work critically for important intellectual content. Goran Šimić: interpretation of data for the work; revising the work critically for important intellectual content. Mirta Boban: conception and design of the work; interpretation of data; drafting the work, revising the work critically for important intellectual content.

## CONFLICT OF INTEREST STATEMENT

The authors declare no conflict of interest.

## ETHICS STATEMENT

The ethical statement is not required.

## Supporting information


Appendix S1.

## Data Availability

The data that support the findings of this study are available from the corresponding author upon reasonable request.
