# Supplementary material for: Effects of heterologous human tau protein expression in yeast models of proteotoxic stress response
Source: CNS Neurosci Ther. 2023 Jun 21;30(6):e14304. doi: 10.1111/cns.14304 (PMC11163194; doi:10.1111/cns.14304)
Supplement: Supplementary file 1 — Appendix S1. [file CNS-30-e14304-s001.docx]

**SUPPORTING INFORMATION**

**Yeast strain construction**

BY4741_Nup49_mScarlet strain was constructed by transforming BY4741 with PCR product amplified from plasmid pFA6a-link-ymScarletI-URA3 using primers prKZ129 and pKZ130, followed by selection for Ura+ colonies.

**Plasmid construction**

Constructs encoding NanoBiT fusion proteins tau-HA-Gly-Ser-SmBiT and tau-V5-Gly-Ser-LgBiT (pKZ04-pKZ08; pKZ035) were constructed in the following way. The sequence of SmBiT and LgBiT were optimized for expression in *S. cerevisiae* by replacing rarely used codons from the original sequences with more commonly used codons in yeast (see sequences below). To be able to distinguish the expression ot tau-SmBiT and tau-LgBiT fusions, constructs were tagged with different epitope tags: tau-SmBiT with HA and tau-LgBiT with V5. Repeats of Gly-Ser were inserted between the epitope tags and the C-terminally linked subunits. Each fusion was placed under the control of the constitutively active *TDH3* gene promoter and, where co-expression from a single plasmid was used (pKZ08 and pKZ35), the constructs were integrated into a common vector in antiparallel directions. Constructs encoding NanoBiT fusion proteins were terminating with short synthetic terminators Tsynth8 (tau-SmBiT) or Tsynth27 (tau-LgBiT)^1^.

The cDNA sequence of the 2N4R isoform of the wild-type human tau protein in plasmids pKZ10-pKZ12, pKZ24, pKZ37, pKZ41, pKZ51 was optimized for expression in yeast by replacing the least frequently used codons with more frequent ones (see sequences below).

Sequences of primers used for the construction of plasmids are listed in the **Table S1** below.

Plasmid pKZ04 was constructed in several steps: first, the DNA fragment “A” was amplified by PCR from pCA1016 using primers prKZ09–10, DNA fragment “B” was synthesized (Eurofins Genomics, Ebersberg Germany) and the DNA fragment “C” was constructed by assembly PCR using primers prKZ26-31. The final plasmid was obtained by homologous recombination in yeast, by cotransformation of fragments A, B and C together with NotI/BamHI-cut pRS316 into a Ura− yeast strain, followed by selection for Ura+ colonies. pKZ05 was created by ligating a large fragment from NotI/BamHI-cut pRS316 with the small fragment of similarly cut pKZ04. pKZ06 was constructed in the following steps: a DNA fragment “A” was amplified by PCR from pCA1016 using primers prKZ10 and prKZ11. DNA fragments B and C were synthesized (Eurofins Genomics, Ebersberg Germany). Plasmid pKZ06 was then created by cotransforming a Ura− yeast strain with EcoRI/XhoI-cut pRS316 and DNA fragments A, B and C, followed by selection for Ura+ colonies. pKZ07 was created by ligating a large fragment from SacI/EagI -cut pRS316 with the small fragment of similarly cut pKZ06. pKZ08 was created by ligating EcoRI/XhoI-cut pKZ04 with similarly cut pKZ06. pKZ10 was created by cotransforming Leu− strain with AvrII/XhoI-cut pXP731and DNA fragment amplified from pKZ05 using primers prKZ19 and prKZ20, followed by selection for Leu+ colonies. pKZ11 was created by ligating a large fragment from AvrII/XhoI cut pXP732 with the small fragment of similarly cut pKZ10. pKZ12 was created by cotransforming Ura− strain with XhoI/SpeI-cut pXP722 and DNA fragment amplified from pKZ10 using primers prKZ20 and prKZ43, followed by selection for Ura+ colonies. pKZ15 was created by ligating EcoRI/KpnI-cut pRS316 with synthesized DNA fragment (Eurofins Genomics, Ebersberg Germany). pKZ24 was created by ligating SalI/BamHI-cut pMB152 with similarly cut PCR product amplified from pKZ19 using primers prKZ72 and prKZ81. pKZ35 was created by ligating SpeI/NotI-cut pKZ06 with similarly cut synthesized DNA fragment. pKZ37 was created by ligating a large fragment from XhoI/KpnI-cut pKZ24 with the small fragment of similary cut pMB211. pKZ41 was created by ligating large fragment from SpeI/AvrII-cut pKZ10 with similarly cut DNA amplified from genomic DNA using primers prKZ105 and prKZ106. pKZ51 was created by cotransforming AvrII-cut pKZ41 and similarly cut DNA fragment amplified from pMB292 using primers prKZ135 and prKZ136.

**Sequence of the gene encoding human tau protein:**

ATGGCTGAGCCCCGTCAGGAGTTCGAAGTGATGGAAGATCACGCTGGGACGTACGGGTTGGGGGACAGAAAAGATCAGGGGGGCTACACCATGCACCAAGACCAAGAGGGTGACACTGACGCTGGCCTGAAAGAATCTCCCCTGCAGACCCCCACTGAGGACGGATCTGAGGAACCAGGCTCTGAAACCTCTGATGCTAAGAGCACTCCAACAGCTGAAGATGTGACAGCACCCTTAGTGGATGAGGGAGCTCCCGGCAAGCAGGCTGCCGCTCAGCCCCACACTGAGATCCCAGAAGGAACCACAGCTGAAGAAGCAGGCATTGGAGACACCCCCAGCCTGGAAGACGAAGCTGCTGGTCACGTGACCCAAGCTCGTATGGTCAGTAAAAGCAAAGACGGGACTGGAAGCGATGACAAAAAAGCCAAGGGGGCTGATGGTAAAACTAAGATCGCCACACCACGTGGAGCAGCCCCTCCAGGCCAGAAGGGCCAGGCCAACGCCACCAGGATTCCAGCAAAAACCCCACCCGCTCCAAAGACACCACCCAGCTCTGGTGAACCTCCAAAATCAGGGGATCGTAGCGGCTACAGCAGCCCCGGCTCCCCAGGCACTCCCGGCAGCCGTTCCCGTACCCCATCCCTTCCAACCCCACCCACCCGTGAGCCCAAGAAGGTGGCAGTGGTCCGTACTCCACCCAAGTCGCCATCTTCCGCCAAGAGCCGTCTGCAGACAGCCCCCGTGCCCATGCCAGACCTGAAGAATGTCAAGTCCAAGATCGGCTCCACTGAGAACCTGAAGCACCAGCCAGGAGGCGGGAAGGTGCAGATAATTAATAAGAAGCTGGATCTTAGCAACGTCCAGTCCAAGTGTGGCTCAAAGGATAATATCAAACACGTCCCGGGAGGCGGCAGTGTGCAAATAGTCTACAAACCAGTTGACCTGAGCAAGGTGACCTCCAAGTGTGGCTCATTAGGCAACATCCATCATAAACCAGGAGGTGGCCAGGTGGAAGTAAAATCTGAGAAGCTTGACTTCAAGGACAGAGTCCAGTCGAAGATTGGGTCCCTGGACAATATCACCCACGTCCCTGGCGGAGGAAATAAAAAGATTGAAACCCACAAGCTGACCTTCCGTGAGAACGCCAAAGCCAAGACAGACCACGGGGCTGAGATCGTGTACAAGTCGCCAGTGGTGTCTGGGGACACTTCTCCACGTCATCTAAGCAATGTCTCCTCCACCGGCAGCATCGACATGGTAGACTCGCCCCAGCTAGCCACTCTAGCTGACGAGGTGTCTGCCTCCCTGGCCAAGCAGGGTTTG

**LgBiT sequence:**

TCATTAACTATTGATAGTCACACGAAATAACATGGATCCATCAGGAGTAATCAAACGCTCATCAATAATCTTATTACCATTCCACAAAGTACCAGTAACAGTAATCTTCTTACCATCAAAAACAGCAATACCTTCATAAGGACGACCAAAATAATTAAGCATATTAGGAGTAACACCATCAATAACCAAAGTACCATAAGGCAGAATAACTTTGAAGTGATGATCATCAACGGGATAGACCACTTTAAAAACTTCTTCAATTTGAGCCATTTGATCAGCAGATAAACCTTCATAAGGAATAATAACATGAATATCAATCTTCAAAGCATTTTCACCAGATCTCACGATACGCTGGATAGGAGTAACAGACACAGCTAAATTTTGTAATAAGGAGGAAACCCCACCCTGTTCCAGAACTTGATCCAGATTGTAGGCTGCAGTCTGTTCCCAATCACCAACAAAATCTTCTAAAGTAAAAACCAT

**SmBiT sequence:**

GTTACTGGTTACAGATTGTTCGAAGAAATTTTG

**Table S1. Primers used in this study to construct plasmids**

| **Primer** | **Sequence (5’ - 3’)** |
| --- | --- |
| prKZ9 | AATACGACTCACTATAGGGCGAATTGGAGCTCCACCGCGGTGGCGGCCGCCTGCTGTAACCCGTACATG |
| prKZ10 | GTCCCAGCGTGATCTTCCATCACTTCGAACTCCTGACGGGGCTCAGCCATTTTGTTTGTTTATGTGTG |
| prKZ11 | TGGGTACCGGGCCCCCCCTCGAGGTCGACGGTATCGATAAGCTTGATATCCTGCTGTAACCCGTACATG |
| prKZ19 | AAGTCATCGAAATAGATATTAAGAAAAACAAACTGTACAATCAATCAATCAATCATCACATAAACCTAGGATGGCTGAGCCCCGTCAGGA |
| prKZ20 | GCGGATGTGGGGGGAGGGCGTGAATGTAAGCGTGACATAACTAATTACATGACTCGAGTTACTACAAACCCTGCTTGGCCAG |
| prKZ26 | CATGGTAGACTCGCCCCAGCTAGCCACTCTAGCTGACGAGGTGTCTGCCTCCCTGGCCAAG |
| prKZ27 | GCATAGTCGGGGACATCGTAGGGGTACCCACCCAAACCCTGCTTGGCCAGGGAGGCAGAC |
| prKZ28 | CGATGTCCCCGACTATGCATCTTATCCATATGACGTTCCAGATTACGCTAGCGTTACTGGT |
| prKZ29 | GTAAATGAGTTTATATATTATCACAAAATTTCTTCGAACAATCTGTAACCAGTAACGCTAGCGTAATC |
| prKZ30 | GATAATATATAAACTCATTTACTTATGTAGGAATAAAGAGTATCATCTTTCAAATCTAGAACTAGTGG |
| prKZ43 | AAGTATCAACAAAAAATTGTTAATATACCTCTATACTTTAACGTCAAGGAGAAAAAACCACTAGTATGGCTGAGCCCCGTCAGG |
| prKZ72 | CGCTGCAGGTCGACATGGCTGAGCCCCGTCAGGA |
| prKZ81 | GGTGGTGGATCCGCGCCTTATTTGTACAATTCGTCCATTC |
| prKZ129 | ACTGTTTATGGATATCGCTGAGAGAATCGCCGTGTTACATCAAAAAACGAAAACACTGGCATCATTGAGCATAATCGGTGACGGTGCTGGT |
| prKZ130 | TATAAATTACATTTGTACAAGACATTTGTACTTGTTATACGCACTATATAAACTTTCAGGGCGATTTACCAGCAGTATAGCGACCAGCAT |
| prKZ135 | CTTTAGATAATTATTACATTTACATCAATAAGAAATCTCATAAAACAAGTACTGTTTATAAGTCCCTAGGATGGTCTCTAAGGGTGAAG |
| prKZ136 | GATCTTCCATCACTTCGAACTCCTGACGGGGCTCAGCACCAGAAGAACCACCACCACCAGAACCACCCCGCGGCTTGTACAATTCGTCCATAC |

**
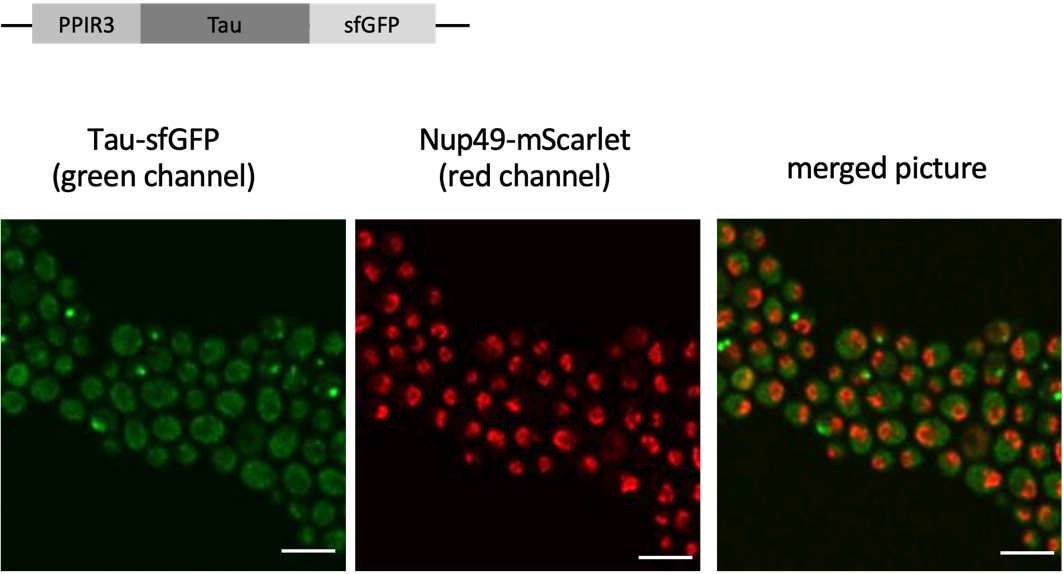
**

**Figure S-1.** **Localization of tau-sfGFP in chronologically aged wild-type cells.** Schematic presentation of PPIR3-tau-sfGFP construct (pKZ37). In 3days old culture of wild type strain (BY4741 transformed with pKZ37), tau-sfGFP formed inclusions in around 10 % of the cells. Scale bar = 10 µm

**Reference**

1. Curran KA, Morse NJ, Markham KA, Wagman AM, Gupta A, Alper HS. Short Synthetic Terminators for Improved Heterologous Gene Expression in Yeast. ACS Synth Biol. 2015 Jul 17;4(7):824-32. doi: 10.1021/sb5003357. Epub 2015 Feb 25. PMID: 25686303.
